# Supplementary material for: Prognosis Risk Model Based on Pyroptosis-Related lncRNAs for Gastric Cancer
Source: Biomolecules. 2023 Mar 3;13(3):469. doi: 10.3390/biom13030469 (PMC10046686; doi:10.3390/biom13030469)
Supplement: Supplementary file 1 [file biomolecules-13-00469-s001.zip › biomolecules-2138697-supplementary.pdf]

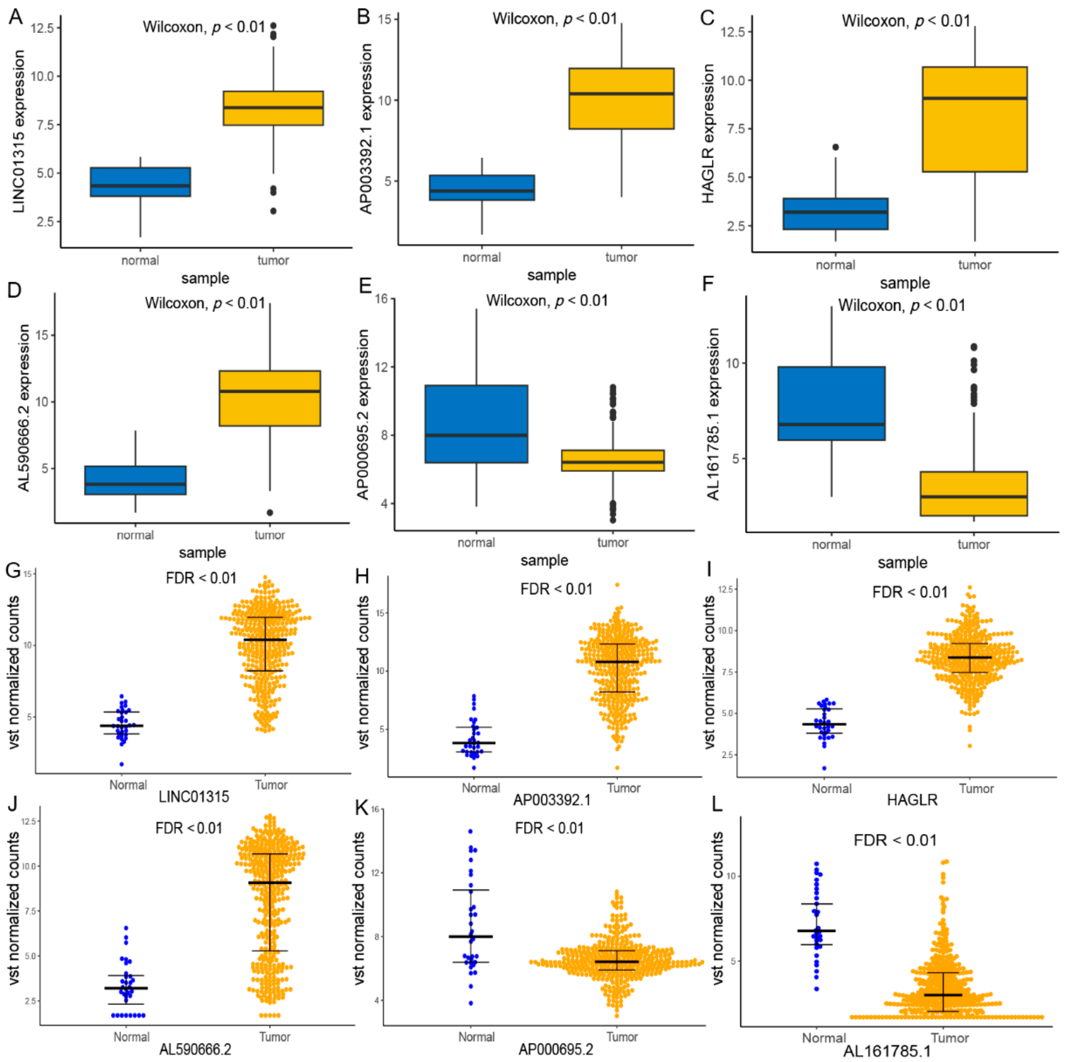

Figure S1. Differential expression of 6LncRNAs in gastric cancer and normal tissues. (**A-F**) TCGA+GTEx dataset, Tumer = 375, Normal = 391. (**G-L**) TCGA dataset, Tumer = 375, Normal = 32. Vst normalized counts: The original count data downloaded by TCGA is used for difference analysis, and the standardized matrix comparison of vst is used. FDR: P value corrected by Benjamini and Hochberg multiple hypothesis test.

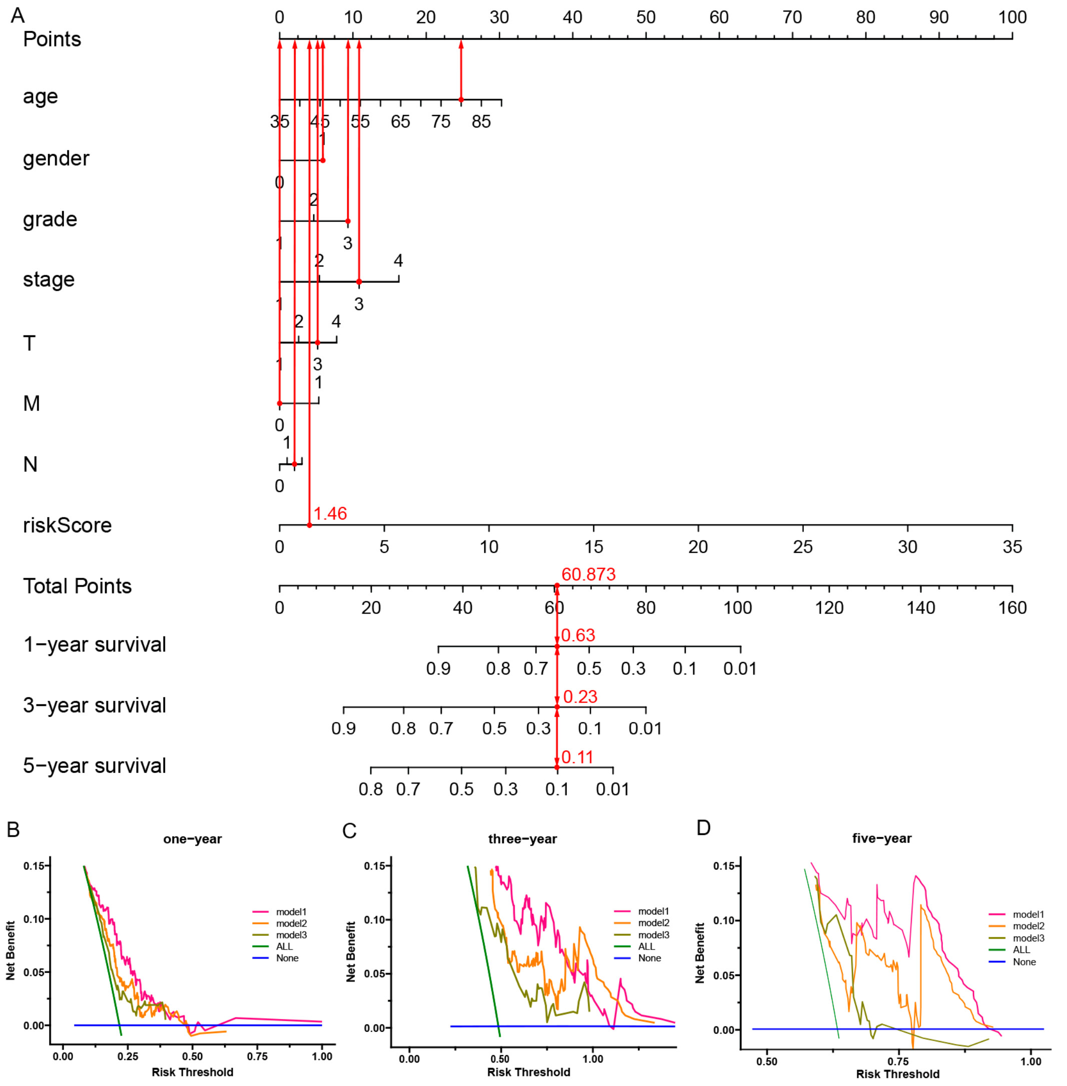

Figure S2: The method of application and effectiveness evaluation of the nomogram. (A) Prediction of survival of random individuals. (B-D) Comparison of the efficacy of the nomogram of the predictive model with conventional prognostic indicators in one -, three -, and five-year survival. Model 1: age + gender + grade + stage + T + M + N + riskScore.
Model 2: age + gender + grade + stage + T + M + N. Model 3: grade + stage + T + M + N

The method of application:

1. The variables in the Nomo map, such as age, sex, tumor grade, stage, and classification, are traditional prognostic factors. The risk score is the individual risk value calculated according to the predictive model, and the corresponding line segment of each variable is marked with a scale, which represents the range of the available value of the variable, and the length of the line segment reflects the contribution of the factor to the survival.
2. Point represents the individual score of each variable under different values, and Total Point represents the total score of the corresponding individual scores of all variables.
3. Outcome：1-, 3-, and 5-year survival.

For example:

The following individuals are randomly simulated. The clinical information is as follows: futime is survival time, unit is day. Fustat is survival state, 0 is alive,1 is dead. Gender: 1 is male, 0 is female. Tumor TMN classification, stage and grade are determined by pathological criteria.

futime	fustat	age	gender	grade	stage	T	M	N	riskScore
385	0	80	1	3	3	3	0	2	1.4662

According to the clinical information and the risk score predicted by the model, the state score of each variable is calculated. Finally, the predicted survival probability is obtained by summing and marking. As shown in the Supplementary Figure 2A, the simulated individual 1-, 3-, and 5-year survival were 0.63, 0.23, and 0.11 respectively.

Decision curve analysis were used to evaluate the effectiveness of prediction (Supplementary Figure 2B-D). The x-axis was determined by the threshold probability, at which the harm of false-positive intervention exceeded the harm of a false-negative non-intervention, and thus an intervention was triggered. The y-axis was a net benefit, which was the relative benefit derived from the proportion of true-positive results subtracted from the proportion of false-positive results weighted by a ratio of threshold probabilities. Under the same probability, the clinical usefulness was better when the net benefit was higher. Model 1 represents the efficiency of the nomogram, model 2 represents the efficiency except the riskScore of the model, and model 3 represents the traditional prognostic interference excluded age and sex. We found that the average net benefit of model 1 is higher than that of model 2 and model 3 at the same time, indicating that the predictive model has clinical potential.
